# Immune responses of *Aedes togoi*, *Anopheles paraliae* and *Anopheles lesteri* against nocturnally subperiodic *Brugia malayi* microfilariae during migration from the midgut to the site of development

**DOI:** 10.1186/s13071-018-3120-1

**Published:** 2018-09-27

**Authors:** Watcharatip Dedkhad, Bruce M Christensen, Lyric C Bartholomay, Deepak Joshi, Chayanit Hempolchom, Atiporn Saeung

**Affiliations:** 1 Graduate PhD’s Degree Program in Parasitology, Faculty of Medicine, Chiang Mai University, Chiang Mai, 50200 Thailand; 2 Department of Pathobiological Sciences, University of Wisconsin, 1656 Linden Drive, Madison, WI 53706 USA; 3 Department of Entomology, University of Kentucky, Lexington, KY 40546-0091 USA; 4 Department of Parasitology, Faculty of Medicine, Chiang Mai University, Chiang Mai, 50200 Thailand

**Keywords:** *Aedes togoi*, *Anopheles paraliae*, *Anopheles lesteri*, *Brugia malayi*, Melanisation, Degeneration

## Abstract

**Background:**

Lymphatic filariasis is a mosquito-borne disease caused by filarioid nematodes. A comparative understanding of parasite biology and host-parasite interactions can provide information necessary for developing intervention programmes for vector control. Here, to understand such interactions, we choose highly susceptible filariasis vectors (*Aedes togoi* and *Anopheles lesteri*) as well as *Anopheles paraliae*, which has lower susceptibility, infected them with nocturnally subperiodic (NSP) *Brugia malayi* microfilariae (mf) and studied the exsheathment, migration and innate immune responses among them.

**Methods:**

Mosquito-parasite relationships were systematically investigated from the time mf entered the midgut until they reached their development site in the thoracic musculature (12 time points).

**Results:**

Results showed that exsheathment of *B*. *malayi* mf occurred in the midgut of all mosquito species and was completed within 24 h post-blood meal. The migration of *B. malayi* mf from the midgut to thoracic muscles of the highly susceptible mosquitoes *Ae. togoi* and *An. lesteri* was more rapid than in the low susceptibility mosquito, *An. paraliae*. Melanisation and degeneration, two distinct refractory phenotypes, of mf were found in the midgut, haemocoel and thoracic musculature of all mosquito species. Melanisation is a complex biochemical cascade that results in deposition of melanin pigment on a capsule around the worms. Also, some biological environments in the body are inhospitable to parasite development and cause direct toxicity that results in vacuolated or degenerated worms. Even though *Ae. togoi* is highly susceptible to *B. malayi*, melanisation responses against *B*. *malayi* mf were first noted in the haemocoel of *Ae*. *togoi*, followed by a degeneration process. In contrast, in *An*. *lesteri* and *An*. *paraliae*, the degeneration process occurred in the haemocoel and thoracic musculature prior to melanisation responses.

**Conclusion:**

This study provides a thorough description of the comparative pathobiology of responses of mosquitoes against the filarial worm *B. malayi*.

## Background

Lymphatic filariasis (LF) is a mosquito-borne disease that results in chronic medical conditions in 856 million people in 52 countries worldwide [1]. The causative pathogens are three species of filarial parasite, i.e. *Wuchereria bancrofti*, *Brugia malayi* and *Brugia timori*, which are transmitted primarily by *Aedes*, *Anopheles*, *Culex* and *Mansonia* mosquitoes. The Global Programme to Eliminate LF (GPELF) was established to eliminate LF by 2020 [1]. Vector control methods can reduce the transmission of the disease by lowering the vector density in areas actively undergoing mass drug administration (MDA) and in post-MDA areas [2]. Vector surveillance also plays a vital role in preventing the occurring of LF recrudescence. The success of control and surveillance for the vector depend on a clear understanding of the vector species involved in transmission [3].

*Aedes togoi* is a vector of filariasis in China, Japan and Taiwan [4, 5]. In Thailand, *Ae*. *togoi* (Chanthaburi strain) has been reported as a highly susceptible vector to nocturnally subperiodic (NSP) *B*. *malayi* (Narathiwat strain), *W*. *bancrofti* (Tak and Kanchanaburi strains), *Brugia pahangi* (Malaysia strain) and *Dirofilaria immitis* (Chiang Mai strain) [6, 7]. *Anopheles lesteri* is also incriminated as an important vector for malaria parasites in China [8] and is highly susceptible to infection with *Plasmodium vivax* in Korea [9, 10]. Recently, by performing cross-mating experiments between *Anopheles paraliae* (Thailand strain) and *An*. *lesteri* (Korea strain) and comparing sequence for the internal transcribed spacer (ITS2) and cytochrome *c* oxidase subunits 1 and 2, Taai et al. [11] showed that the two species are synonymous. However, remarkable differences were seen in their vector competences as *An*. *lesteri* was highly susceptible to *B*. *malayi* compared to *An*. *paraliae* [12].

Within the mosquito, there are physical and biochemical barriers that affect the compatibility of the vector-pathogen association (vector competence), i.e. cibarial and pharyngeal armature (foregut), midgut, haemolymph and haemocoel, and thoracic musculature [13–15]. In susceptible vectors, microfilariae (mf) circulate in the peripheral blood of infected hosts and are ingested with a blood meal and move through the midgut lumen prior to crossing the midgut epithelium. Then, mf migrate in the mosquito’s haemolymph to access the thoracic musculature and penetrate into the indirect flight muscles, the site of development of worms to the infective, third-stage larvae (L3s) [16].

The midgut is one of the first tissue barriers preventing pathogens from entering the body cavity. Numerous studies have suggested that the exsheathment of mf either occurred exclusively in the lumen of the midgut [17–19] or the haemocoel as well as the midgut lumen [20–22]. Likewise, the study by Chen & Shih [23] demonstrated that the exsheathment of *B. pahangi* microfilariae occurred in the lumen of midgut and haemocoel of susceptible and refractory strains of *Aedes aegypti*. Jariyapan et al. [22] showed the exsheathment of NSP *B*. *malayi* microfilariae happens in the haemocoel of *Ae. togoi*. However, they neither observed the invasion of mf to the thoracic musculature nor the immune response of mosquitoes against mf during migration from the midgut to the development site in their study. By contrast, melanised mf sheaths of *B. pahangi* have been found in the haemocoel of *Ae*. *aegypti* and *Anopheles quadrimaculatus* [24]. Melanised mf sheaths of *B*. *malayi* were observed in the haemocoel of both strains of *An*. *quadrimaculatus* (refractory and susceptible) and the susceptible strain of *Ae*. *aegypti* (Black-eyed, Liverpool) [25].

Unlike competent vectors, some mosquitoes limit filarial worm infections with various refractory or resistance mechanisms. Melanisation is a robust and potent mechanism that can make a mosquito entirely refractory to filarial worms, as is the case in the mosquito, *Armigeres subalbatus*, infected with *B. malayi* [13]. Melanisation is thought to kill pathogens through nutrient starvation and/or direct toxic effects of reaction intermediates and by-products [26]. During the process of melanisation, several free radicals or cytotoxic molecules are generated and released [27]. The direct toxicity effect on *B. malayi* larvae has been noticed in the first-stage larvae (L1) found in the thoracic muscle fibres of mosquitoes with high and low parasite susceptibility [12, 28].

It is well accepted that a better understanding of parasite biology and host-parasite interactions are essential for the development of effective tools or strategies for vector control programmes, particularly the development of genetically modified mosquitoes that are refractory to filarial worm development. We reasoned that a comparative study of exsheathment and melanisation immune responses against *B*. *malayi*, from the time mf enter the midgut until they develop to L3 in the thoracic musculature, would be useful to understand the range of parasite susceptibility and thereby vector competence for three crucial Asian malaria and LF parasite vector species. Therefore, we utilised an interesting host-parasite model based on the highly susceptible strains of *Ae*. *togoi* and *An*. *lesteri*, the low susceptible strain of *An*. *paraliae* and NSP *B*. *malayi*, for systematically investigating exsheathment, migration of mf and host immune responses.

## Methods

### Mosquito species

*Aedes togoi*, *An. lesteri* and *An. paraliae* laboratory mosquito strains were used in this study [29]. All mosquito species were established successfully for many consecutive generations in the insectary of the Department of Parasitology, Faculty of Medicine, Chiang Mai University, Thailand, at 27 ± 2 °C, 70–80% relative humidity, and 12:12 day night ratios adjusted with fluorescent lighting and natural lights coming from the windows [30].

### Maintenance of *B*. *malayi* and preparation of blood containing *B. malayi* microfilariae

Mongolian jirds (*Meriones unguiculatus*) were used for maintaining the NSP *B*. *malayi*, that originated from a resident of the Bang Paw district, Narathiwat Province, South Thailand, at the animal house of the Faculty of Medicine, Chiang Mai University, Chiang Mai, Thailand [31]. We followed the systematic procedures as described by Saeung & Choochote [32] for parasite maintenance. This provides a simple system for maintenance and mass production of *B*. *malayi* in a space-constrained laboratory.

### Infection of mosquitoes with *B. malayi* microfilariae

Five-day-old adult female *Ae. togoi*, *An*. *lesteri* and *An. paraliae* were starved for 24 h and allowed to feed on human blood, which was taken from the principal investigator, containing *B. malayi* mf (average microfilarial density of 330 mf/20 μl for three experiments) through an artificial membrane [32]. We allowed mosquitoes to feed on the *B. malayi*-infected blood meal (mixed well) and five fully engorged mosquitoes were randomly selected for dissection. The size of the blood meal did not differ among the three species as seen in our previous study by Dedkhad et al. [29]. We found that the average number of mf per infected midgut dissected immediately after feeding on blood containing *B*. *malayi* microfilariae ranged between 1–299 mf for *Ae*. *togoi* and 5–245 mf for *An*. *paraliae*.

### Exsheathment and migration studies of NSP *B*. *malayi* mf

Infected adult females of *Ae. togoi*, *An. lesteri*, and *An. paraliae* were dissected after full engorgement and their midguts were removed in normal saline. Five mosquitoes were dissected on glass slides with sodium chloride solution 0.85% (Sigma-Aldrich, Queenstown, Singapore) at different time points (5 min, and 1, 2, 3, 4, 5, 6, 12, 18, 24, 48 and 72 h, *n* = 60). Each dissected midgut was transferred to a new glass slide. Mosquitoes were discarded if the gut ruptured during dissection. The remaining mosquito tissues (thorax and abdomen with fluids) were dissected separately on a glass slide. These samples were then made into thick blood films, dried, de-hemoglobinized, fixed with absolute methanol, and stained with Giemsa (pH 7.2). The number and percentage of sheathed and exsheathed mf of *B*. *malayi* from the midgut (ingested), haemocoel and thoracic muscle fibres of each mosquito species were counted at different time points. Photographs of the mf were taken using a digital camera attached to a compound microscope (BX53, Olympus®, Japan).

### Mosquito immune responses against filarial worms

The number and percentage of normal and abnormal (degenerated and melanised) mf from the midgut, haemocoel and thoracic muscle fibres of each mosquito species, detected on the slide containing normal saline, were counted at different time points in the drop of normal saline. Photographs of the mf were taken using the same microscope system. The mf were counted and scored as “normal” if mf were alive with intact morphology and had normal movement, and as “melanised” if mf had evidence of a melanin capsule and had partial movement or were immotile (in case of completed melanised), and as “degenerated” if mf had vacuolated internal organs without any evidence of melanisation and had sluggish movement or were immotile [33].

### Data analysis

The mean total number of normal and abnormal mf recovered from all mosquito species were compared using non-parametric Kruskal-Wallis tests. A *post-hoc* Dunn’s test was used for multiple comparisons of means. The level of significance was set at 5% (*P*-value < 0.05). Statistical analyses were conducted using IBM SPSS statistics, version 22 for Windows (Chicago, SPSS Inc.). The level of significance was set at 5% (*P*-value < 0.05).

## Results

### Exsheathment and migration of *B*. *malayi* mf in *Ae. togoi*, *An. lesteri* and *An. paraliae*

**Fig. 1:**
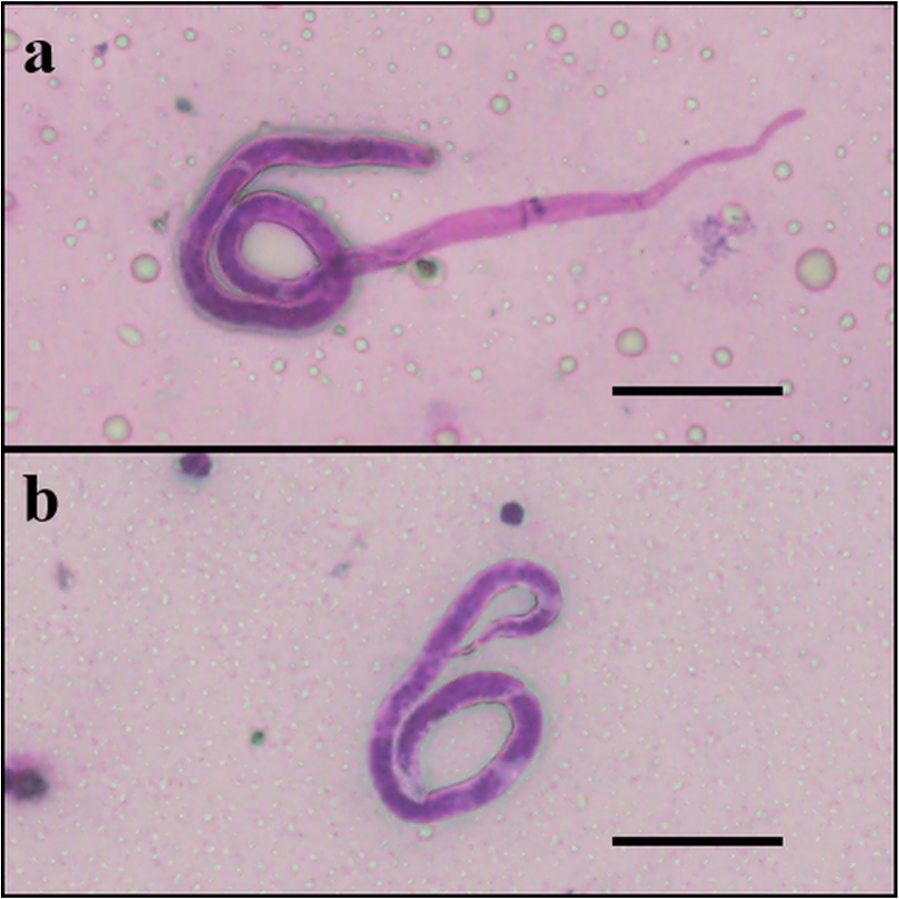
*Brugia* *malayi* microfilariae with intact morphology in the midgut of *Ae*. *togoi*. **a** A sheathed microfilaria. **b** An exsheathed microfilaria. *Scale-bars*: 50 μm

The mean number of total microfilariae (mf), sheathed (Fig. 1a) and exsheathed (Fig. 1b) per body region (midgut, haemocoel and thorax) found in the three mosquito species are given in Tables 1, 2 and 3. Of the 12 time points, the mean total number of mf found in the midgut of *Ae*. *togoi*, *An*. *lesteri* and *An*. *paraliae* ranged between 0.2–43.4, 0.2–99.8 and 0.2–36.2, respectively, for three experiments. The mf penetrated the midgut wall into the haemocoel and invaded the thoracic muscle fibres of *Ae*. *togoi* as soon as 5 min after the blood meal, while mf were first seen in the thoracic muscle fibres of *An*. *paraliae* and *An*. *lesteri* at 1 and 2 h post-feeding, respectively. More than 50% of exsheathed mf were found in the midgut of *Ae*. *togoi* at 5 min post-blood meal, whereas 20% of exsheathed mf were found in those of *An*. *lesteri* and *An*. *paraliae*. All ingested mf were completely exsheathed in the midgut at 12 h for *Ae*. *togoi* and *An*. *lesteri*, and at 18 h post-blood meal for *An*. *paraliae*. Importantly, all mf found in the haemocoel and the thoracic muscle fibres of three species were exsheathed.

**Table 1:** Mean number of sheathed and exsheathed mf of *B. malayi* from the midgut, haemocoel (haemolymph) and thoracic musculature of *Ae. togoi* at different time points post-infection (PI)

Time PI	Mean no. of total mf per infected midgut (range)	Mean no. of total mf per infected haemocoel (range)	Mean no. of total mf per infected thoracic muscles (range)	Sheathed and exsheathed mf found in each part of mosquitoes^a^					
				Midgut		Haemocoel		Thorax	
				Mean no. of sheathed mf (%)	Mean no. of exsheathed mf (%)	Mean no. of sheathed mf (%)	Mean no. of exsheathed mf (%)	Mean no. of sheathed mf (%)	Mean no. of exsheathed mf (%)
5 min	43.4 (10–92)	3 (0–5)	0.4 (0–1)	15.6 (36)	27.8 (64)	0.4 (13)	2.6 (87)	0 (0)	0.4 (100)
1 h	20.8 (10–24)	4.2 (0–13)	0.8 (0–2)	4 (19)	16.8 (81)	0 (0)	4.2 (100)	0 (0)	0.8 (100)
2 h	20.6 (11–36)	3.2 (1–9)	2.8 (1–5)	4.2 (20)	16.4 (80)	0 (0)	3.2 (100)	0 (0)	2.8 (100)
3 h	16.4 (9–26)	5.8 (2–12)	5 (3–8)	3 (18)	13.4 (82)	0 (0)	5.8 (100)	0 (0)	5 (100)
4 h	35.6 (11–74)	9.6 (3–20)	9 (4–15)	1.4 (4)	34.2 (96)	0 (0)	9.6 (100)	0 (0)	9 (100)
5 h	42.6 (19–84)	10.2 (5–15)	24.4 (16–35)	1.2 (3)	41.4 (97)	0 (0)	10.2 (100)	0 (0)	24.4 (100)
6 h	10 (0–18)	5.6 (0–17)	8.4 (0–21)	0.4 (4)	9.6 (96)	0 (0)	5.6 (100)	0 (0)	8.4 (100)
12 h	7 (0–18)	1.4 (0–6)	6 (0–19)	0 (0)	7 (100)	0 (0)	1.4 (100)	0 (0)	6 (100)
18 h	14.6 (4–38)	3.6 (1–8)	14.6 (3–43)	0 (0)	14.6 (100)	0 (0)	3.6 (100)	0 (0)	14.6 (100)
24 h	5.6 (2–9)	3.6 (0–12)	12.8 (1–22)	0.2 (4)	5.4 (96)	0 (0)	3.6 (100)	0 (0)	12.8 (100)
48 h	0.2 (0–1)	5.2 (1–9)	21.4 (16–47)	0 (0)	0.2 (100)	0 (0)	5.2 (100)	0 (0)	21.4 (100)
72 h	0.2 (0–1)	1.8 (1–5)	13.6 (0–30)	0 (0)	0.2 (100)	0 (0)	1.8 (100)	0 (0)	13.6 (100)

^a^Dissected from five infected mosquitoes per time point

**Table 2:** Mean number of sheathed and exsheathed mf of *B. malayi* from the midgut, haemocoel (haemolymph) and thoracic musculature of *An. lesteri* at different time points post-infection (PI)

Time PI	Mean no. of total mf per infected midgut (range)	Mean no. of total mf per infected haemocoel (range)	Mean no. of total mf per infected thoracic muscles (range)	Sheathed and exsheathed mf found in each part of mosquitoes^a^					
				Midgut^a^		Haemocoel^a^		Thorax^a^	
				Mean no. of sheathed mf (%)	Mean no. of exsheathed mf (%)	Mean no. of sheathed mf (%)	Mean no. of exsheathed mf (%)	Mean no. of sheathed mf (%)	Mean no. of exsheathed mf (%)
5 min	77 (34–166)	0.2 (0–1)	–	65 (84)	12 (16)	0 (0)	0.2 (100)	0 (0)	0 (0)
1 h	99.8 (32–242)	13.6 (2–31)	3.8 (0–9)	37.8 (38)	62 (62)	0 (0)	13.6 (100)	0 (0)	3.8 (100)
2 h	57.8 (23–89)	9.8 (3–14)	11.4 (3–20)	12 (21)	45.8 (79)	0 (0)	9.8 (100)	0 (0)	11.4 (100)
3 h	77.2 (13–254)	13.4 (1–37)	10.8 (5–19)	6.2 (8)	71 (92)	0 (0)	13.4 (100)	0 (0)	10.8 (100)
4 h	26.8 (15–38)	5.6 (3–11)	12.8 (6–23)	2.8 (10)	24 (90)	0 (0)	5.6 (100)	0 (0)	12.8 (100)
5 h	27.6 (14–50)	10.2 (1–37)	25.6 (9–64)	2.6 (9)	25 (91)	0 (0)	10.2 (100)	0 (0)	25.6 (100)
6 h	21.6 (13–32)	7.8 (2–17)	22.2 (4–33)	0.4 (2)	21.2 (98)	0 (0)	7.8 (100)	0 (0)	22.2 (100)
12 h	26.4 (20–37)	1.8 (0–3)	19.4 (10–31)	0 (0)	26.4 (100)	0 (0)	1.8 (100)	0 (0)	19.4 (100)
18 h	17 (6–28)	0.8 (0–2)	16.6 (10–25)	0 (0)	17 (100)	0 (0)	0.8 (100)	0 (0)	16.6 (100)
24 h	14.6 (9–20)	0.8 (0–3)	17.2 (9–31)	0 (0)	14.6 (100)	0 (0)	0.8 (100)	0 (0)	17.2 (100)
48 h	1.6 (0–4)	0.4 (0–2)	17.2 (12–20)	0 (0)	1.6 (100)	0 (0)	0.4 (100)	0 (0)	17.2 (100)
72 h	0.2 (0–1)	2 (0–5)	20.8 (13–39)	0 (0)	0.2 (100)	0 (0)	2 (100)	0 (0)	20.8 (100)

^a^Dissected from five infected mosquitoes per time point

**Table 3:** Mean number of sheathed and exsheathed mf of *B. malayi* from the midgut, haemocoel (haemolymph) and thoracic musculature of *An. paraliae* at different time points post-infection (PI)

Time PI	Mean no. of total mf per infected midgut (range)	Mean no. of total mf per infected haemocoel (range)	Mean no. of total mf per infected thoracic muscles (range)	Sheathed and exsheathed mf found in each part of mosquitoes^a^					
				Midgut^a^		Haemocoel^a^		Thorax^a^	
				Mean no. of sheathed mf (%)	Mean no. of exsheathed mf (%)	Mean no. of sheathed mf (%)	Mean no. of exsheathed mf (%)	Mean no. of sheathed mf (%)	Mean no. of exsheathed mf (%)
5 min	36.2 (6–83)	0 (0)	0 (0)	29.2 (81)	7 (19)	0 (0)	0 (0)	0 (0)	0 (0)
1 h	25 (13–46)	0 (0)	0 (0)	8.6 (34)	16.4 (66)	0 (0)	0 (0)	0 (0)	0 (0)
2 h	15.8 (5–20)	2.2 (1–4)	2.2 (0–4)	3.8 (24)	12 (76)	0 (0)	2.2 (100)	0 (0)	2.2 (100)
3 h	13.8 (8–24)	0.4 (0–1)	2.4 (0–5)	3.2 (23)	10.6 (77)	0 (0)	0.4 (100)	0 (0)	2.4 (100)
4 h	23.2 (19–30)	2 (0–4)	3.2 (2–4)	2.8 (12)	20.4 (88)	0 (0)	2 (100)	0 (0)	3.2 (100)
5 h	28.8 (4–90)	0.8 (0–3)	8 (4–14)	2.6 (9)	26.2 (91)	0 (0)	0.8 (100)	0 (0)	8 (100)
6 h	15 (8–31)	0.6 (0–2)	5 (2–8)	0.6 (4)	14.4 (96)	0 (0)	0.6 (100)	0 (0)	4.2 (100)
12 h	18.4 (4–43)	5.2 (0–12)	16 (3–39)	0.4 (2)	18 (98)	0 (0)	5.2 (100)	0 (0)	16 (100)
18 h	7.4 (1–18)	1.2 (0–2)	11.2 (0–26)	0 (0)	7.4 (100)	0 (0)	1.2 (100)	0 (0)	11.2 (100)
24 h	1.6 (0–3)	0.2 (0–1)	5.8 (4–7)	0 (0)	1.6 (100)	0 (0)	0.2 (100)	0 (0)	5.8 (100)
48 h	0.6 (0–3)	0 (0)	5.6 (1–10)	0 (0)	0.6 (100)	0 (0)	0 (0)	0 (0)	5.6 (100)
72 h	0.2 (0–1)	0 (0)	6.6 (1–13)	0 (0)	0.2 (100)	0 (0)	0 (0)	0 (0)	6.6 (100)

^a^Dissected from five infected mosquitoes per time point

### Investigation of host immune responses against *B. malayi* microfilariae

To investigate the effect of host immune response on *B. malayi* mf, the midgut, haemocoel and thoracic muscle fibres were examined using light microscopy. The mean total number of normal and abnormal (degenerated and melanised) mf recovered from all parts were compared among three mosquito species (Table 4). There were significant differences in mean numbers of normal mf observed at four time points including between *Ae. togoi* and *An. paraliae* at 1 h (Dunn’s test, *P* = 0.033), *Ae. togoi* and *An. lesteri* at 18 h (Dunn’s test*, P* = 0.021) and 24 h (Dunn’s test, *P* = 0.013), and *An. lesteri* and *An. paraliae* (Dunn’s test, *P* = 0.04) at 72 h. The higher number of abnormal mf (degeneration and melanisation) was observed in the low susceptible mosquito, *An*. *paraliae*, than those of both high susceptible mosquitoes. A significant difference in the mean numbers of abnormal mf was found between *Ae. togoi* and *An. paraliae* at 12 h (Dunn’s test, *P* = 0.005), 18 h (Dunn’s test, *P* = 0.005) and 24 h (Dunn’s test*, P* = 0.01) (Table 4).

**Table 4:** Comparisons of average numbers of normal and abnormal mf (degeneration and melanisation) of *B. malayi* from three parts [the midgut, haemocoel (haemolymph) and thoracic musculature] of *Ae. togoi* (AT), *An*. *lesteri* (AL) and *An*. *paraliae* (AP) at different time points post-infection (PI)

Time PI	Strain	Total no. of mf^a^	No. of normal mf	*H*-value^b^	*P*-value^c^	No. of abnormal mf	*H*-value^b^	*P*-value^c^
5 min	AT	132	131	0.539		1	nd	
	AL	194	194			0		
	AP	161	161			0		
1 h	AT	270	270	0.03	AP *vs* AT, *P* = 0.033	0	nd	
	AL	256	256			0		
	AP	100	100			0		
2 h	AT	97	97	0.074		0	nd	
	AL	244	243			1		
	AP	105	105			0		
3 h	AT	186	182	0.285		4	0.178	
	AL	338	338			0		
	AP	182	178			4		
4 h	AT	188	187	0.18		1	0.162	
	AL	199	198			1		
	AP	130	124			6		
5 h	AT	222	220	0.364		2	0.461	
	AL	313	309			4		
	AP	131	123			8		
6 h	AT	88	87	0.571		1	0.182	
	AL	311	306			5		
	AP	111	102			9		
12 h	AT	72	69	0.146		3	0.005	AT *vs* AP, *P* = 0.005
	AL	173	165			8		
	AP	113	82			31		
18 h	AT	19	19	0.02	AT *vs* AL, *P* = 0.021	0	0.006	AT *vs* AP, *P* = 0.005
	AL	298	282			16		
	AP	254	215			39		
24 h	AT	24	22	0.017	AT *vs* AL, *P* = 0.013	2	0.002	AT *vs* AP, *P* = 0.01
	AL	158	143			15		
	AP	132	64			68		
48 h	AT	72	57	0.092		15	0.165	
	AL	38	21			17		
	AP	44	11			33		
72 h	AT	74	24	0.043	AP *vs* AL, *P* = 0.04	50	0.52	
	AL	92	64			28		
	AP	39	10			29		

^a^Dissected from five infected mosquitoes per time point

^b^Kruskal-Wallis test

^c^Dunn’s test

*Abbreviation*: *nd* not done

**Fig. 2:**
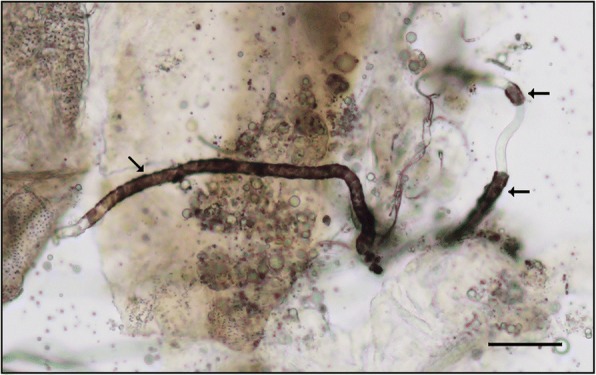
Melanised microfilariae recovered from the thoracic muscle fibres of *Ae*. *togoi* at 12 h post-blood meal. Arrows indicate the partially melanised capsule of the microfilaria. *Scale-bar*: 20 μm

**Fig. 3:**
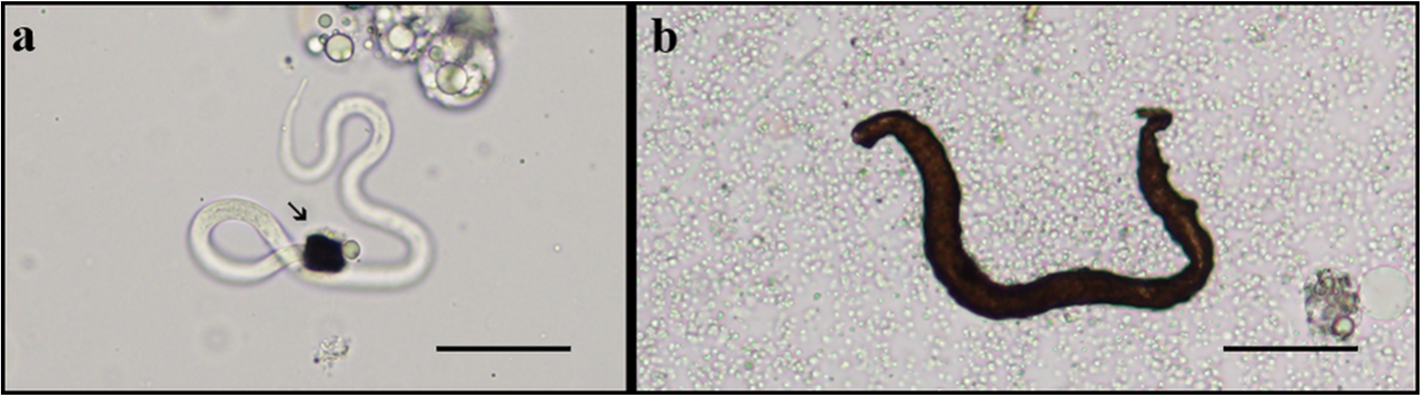
Melanised microfilariae recovered from the haemocoel of *An*. *paraliae*. **a** Melanised microfilaria at 4 h post-blood meal (arrow indicates the partial melanin capsule around the cephalic region of the microfilaria). **b** Completely melanised capsule of the microfilaria at 48 h post-blood meal. *Scale-bars*: 20 μm

**Fig. 4:**
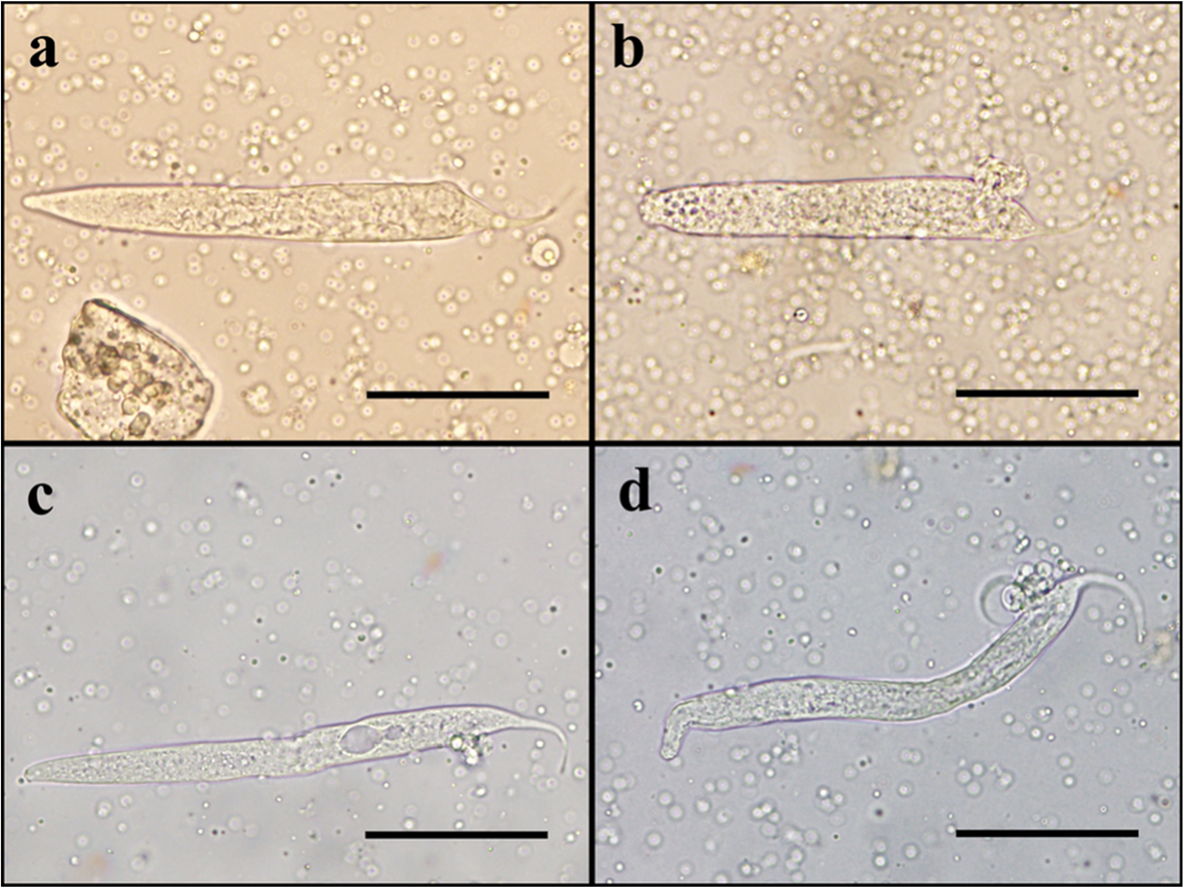
First-stage larvae recovered from thoracic muscle fibres of *An*. *lesteri* and *An*. *paraliae* at 72 h post-blood meal. **a** Normal live larva with intact cuticle recovered from *An*. *lesteri*. **b** Larva with abnormal shape recovered from *An*. *lesteri*. **c** Vacuolated larva recovered from *An*. *paraliae.* **d** Larva with abnormal shape recovered from *An*. *paraliae*. *Scale-bars*: 20 μm

Details of the mean number of normal and abnormal (degenerated or melanised) mf per midgut, haemocoel, and thoracic muscle fibres are shown in Tables 5, 6 and 7. The majority of mf recovered from all mosquito species showed normal morphology. Degenerated mf were found only in the midgut at 4–5 h and thoracic muscle fibres at 48 and 72 h of *Ae*. *togoi*. For *An*. *lesteri*, degenerating mf were found in all parts with lower numbers but earlier (at 2 h in thoracic muscle fibres) than melanised mf. The earliest time point at which degenerated mf were found in the midgut and haemocoel of *An*. *paraliae* was 3 h post-blood meal. In *Ae*. *togoi*, melanised mf were observed in the haemocoel and the thoracic muscle fibres at several time points post-blood meal (Fig. 2). Melanised mf were found in the haemocoel and the thoracic muscle fibres of *An*. *lesteri* beginning at 5 h post-blood meal, whereas these mf were observed in haemocoel of *An*. *paraliae* at 4 h post-blood meal (Fig. 3a). The higher percentages of melanised mf than degenerated mf were found in the haemocoel of *An*. *paraliae* at almost all time points (Fig. 3b), except at 12 and 18 h post-blood meal. However, the percentage of degenerated mf found in the thoracic muscle fibres showed higher levels of degenerated mf at all time points. Also, normal (Fig. 4a) and abnormal (Fig. 4b) L1s were also found in the thoracic muscle fibres of *An*. *lesteri* at 72 h post-blood meal. The abnormal L1s were found in the thoracic muscle fibres of *An*. *paraliae* at 72 h post-blood meal (Fig. 4c, d).

**Table 5:** Distribution of normal and abnormal mf (degeneration and melanisation) of *B. malayi* from the midgut, haemocoel (haemolymph) and thoracic musculature of *Ae. togoi* at different time points post-infection (PI)

Time PI	Mean no. of total mf per infected midgut (range)	Mean no. of total mf per infected haemocoel (range)	Mean no. of total mf per infected thorax (range)	Normal and abnormal mf found in each part of mosquitoes^a^								
				Midgut^a^			Haemocoel^a^			Thorax^a^		
				Mean no. of normal mf (%)	Mean no. of degenerated mf (%)	Mean no. of melanised mf (%)	Mean no. of normal mf (%)	Mean no. of degenerated mf (%)	Mean no. of melanised mf (%)	Mean no. of normal mf (%)	Mean no. of degenerated mf (%)	Mean no. of melanised mf (%)
5 min	25.2 (12–36)	1.2 (2–4)	0 (0)	25.2 (100)	0 (0)	0 (0)	1 (83)	0 (0)	0.2 (17)	0 (0)	0 (0)	0 (0)
1 h	48.4 (25–72)	3.6 (1–7)	2 (1–10)	48.4 (100)	0 (0)	0 (0)	3.6 (100)	0 (0)	0 (0)	2 (100)	0 (0)	0 (0)
2 h	9.2 (3–16)	5.6 (3–8)	4.6 (2–9)	9.2 (100)	0 (0)	0 (0)	5.6 (100)	0 (0)	0 (0)	4.6 (100)	0 (0)	0 (0)
3 h	14.2 (11–18)	8.8 (0–10)	14.2 (0–19)	14.2 (100)	0 (0)	0 (0)	8.2 (93)	0 (0)	0.6 (7)	14 (99)	0 (0)	0.2 (1)
4 h	15.6 (5–21)	8.8 (2–12)	13 (8–21)	15.6 (99)	0.2 (1)	0 (0)	8.8 (100)	0 (0)	0 (0)	13 (100)	0 (0)	0 (0)
5 h	21.4 (0–44)	8.2 (0–15)	14.8 (13–26)	21.2 (99)	0.2 (1)	0 (0)	8 (98)	0 (0)	0.2 (2)	14.8 (100)	0 (0)	0 (0)
6 h	6.6 (0–15)	3.4 (0–5)	7.6 (0–17)	6.6 (100)	0 (0)	0 (0)	3.2 (94)	0 (0)	0.2 (6)	7.6 (100)	0 (0)	0 (0)
12 h	1.4 (0–4)	1.6 (0–4)	11.4 (5–20)	1.4 (100)	0 (0)	0 (0)	1 (62)	0 (0)	0.6 (38)	11.4 (100)	0 (0)	0 (0)
18 h	0 (0)	0.8 (0–3)	3.2 (0–12)	0 (0)	0 (0)	0 (0)	0.6 (75)	0 (0)	0.2 (25)	3.2 (100)	0 (0)	0 (0)
24 h	0 (0)	0.2 (0–1)	4.6 (0–13)	0 (0)	0 (0)	0 (0)	0 (0)	0 (0)	0.2 (100)	4.4 (96)	0 (0)	0.2 (4)
48 h	0 (0)	0.4 (0–1)	14 (0–30)	0 (0)	0 (0)	0 (0)	0.2 (50)	0 (0)	0.2 (50)	11.2 (80)	1.8 (13)	1 (7)
72 h	0 (0)	5.2 (0–12)	9.6 (0–15)	0 (0)	0 (0)	0 (0)	0 (0)	0 (0)	5.2 (100)	4.8 (50)	2.6 (27)	2.2 (23)

^a^Dissected from five infected mosquitoes per time point

**Table 6:** Distribution of normal and abnormal mf (degeneration and melanisation) of *B. malayi* from the midgut, haemocoel (haemolymph) and thoracic musculature of *An. lesteri* at different time points post-infection (PI)

Time PI	Mean no. of total mf per infected midgut (range)	Mean no. of total mf per infected haemocoel (range)	Mean no. of total mf per infected thorax (range)	Normal and abnormal mf found in each part of mosquitoes^a^								
				Midgut^a^			Hemocoel^a^			Thorax^a^		
				Mean no. of normal mf (%)	Mean no. of degenerated mf (%)	Mean no. of melanised mf (%)	Mean no. of normal mf (%)	Mean no. of degenerated mf (%)	Mean no. of melanised mf (%)	Mean no. of normal mf (%)	Mean no. of degenerated mf (%)	Mean no. of melanised mf (%)
5 min	38.8 (21–71)	0 (0)	0 (0)	38.8 (100)	0 (0)	0 (0)	0 (0)	0 (0)	0 (0)	0 (0)	0 (0)	0 (0)
1 h	40.2 (24–89)	8.4 (1–25)	2.6 (0–4)	40.2 (100)	0 (0)	0 (0)	8.4 (100)	0 (0)	0 (0)	2.6 (100)	0 (0)	0 (0)
2 h	24.6 (14–38)	12 (0–30)	12.2 (0–18)	24.6 (100)	0 (0)	0 (0)	12 (100)	0 (0)	0 (0)	12 (98)	0.2 (2)	0 (0)
3 h	31.4 (18–60)	18.4 (4–39)	17.8 (10–34)	31.4 (100)	0 (0)	0 (0)	18.4 (100)	0 (0)	0 (0)	17.8 (100)	0 (0)	0 (0)
4 h	15.6 (5–28)	10.2 (0–24)	14 (2–21)	15.6 (100)	0 (0)	0 (0)	10 (98)	0.2 (2)	0 (0)	14 (100)	0 (0)	0 (0)
5 h	27.4 (6–83)	9.4 (0–28)	25.8 (0–78)	27.4 (100)	0 (0)	0 (0)	9 (96)	0 (0)	0.4 (4)	25.4 (98)	0 (0)	0.4 (2)
6 h	21.6 (6–48)	13.6 (0–61)	27 (0–79)	21.6 (100)	0 (0)	0 (0)	13.2 (97)	0 (0)	0.4 (3)	26.4 (98)	0 (0)	0.6 (2)
12 h	4.4 (0–13)	3.2 (0–9)	27 (0–44)	4.4 (100)	0 (0)	0 (0)	2.4 (74)	0.4 (13)	0.4 (13)	26.2 (98)	0.4 (1)	0.4 (1)
18 h	12.6 (0–29)	4.2 (0–5)	42.8 (0–95)	12.4 (98)	0.2 (2)	0 (0)	2.4 (57)	0.8 (19)	1 (24)	41.6 (97)	1 (2)	0.2 (1)
24 h	7 (4–10)	2.8 (0–4)	21.8 (0–31)	7 (100)	0 (0)	0 (0)	0.6 (21)	0.2 (7)	2 (72)	21 (96)	0.6 (3)	0.2 (1)
48 h	1.2 (0–3)	1.2 (0–2)	5.2 (0–9)	0 (0)	1.2 (100)	0 (0)	0 (0)	0 (0)	1.2 (100)	4.2 (80)	0.4 (8)	0.6 (12)
72 h	0.4 (0–2)	2.8 (0–10)	15.2 (0–25)	0 (0)	0.4 (100)	0 (0)	0 (0)	0 (0)	2.8 (100)	12.8 (84)	1.4 (9)	1 (7)

^a^Dissected from five infected mosquitoes per time point

**Table 7:** Distribution of normal and abnormal mf (degeneration and melanisation) of *B. malayi* from the midgut, haemocoel (haemolymph) and thoracic musculature of *An. paraliae* at different time points post-infection (PI)

Time PI	Mean no. of total mf per infected midgut (range)	Mean no. of total mf per infected haemocoel (range)	Mean no. of total mf per infected thorax (range)	Normal and abnormal mf found in each part of mosquitoes^a^								
				Midgut^a^			Hemocoel^a^			Thorax^a^		
				Mean no. of normal mf (%)	Mean no. of degenerated mf (%)	Mean no. of melanised mf (%)	Mean no. of normal mf (%)	Mean no. of degenerated mf (%)	Mean no. of melanised mf (%)	Mean no. of normal mf (%)	Mean no. of degenerated mf (%)	Mean no. of melanised mf (%)
5 min	32.2 (15–49)	0 (0)	0 (0)	32.2 (100)	0 (0)	0 (0)	0 (0)	0 (0)	0 (0)	0 (0)	0 (0)	0 (0)
1 h	17.4 (6–43)	1.4 (0–7)	1.2 (0–2)	17.4 (100)	0 (0)	0 (0)	1.4 (100)	0 (0)	0 (0)	1.2 (100)	0 (0)	0 (0)
2 h	12.8 (5–26)	4 (0–9)	4.2 (2–8)	12.8 (100)	0 (0)	0 (0)	4 (100)	0 (0)	0 (0)	4.2 (100)	0 (0)	0 (0)
3 h	15.6 (0–30)	7.2 (0–24)	13.6 (0–32)	15.2 (97)	0.4 (3)	0 (0)	6.8 (94)	0.4 (6)	0 (0)	13.6 (100)	0 (0)	0 (0)
4 h	11.2 (0–16)	6.8 (0–9)	8 (4–14)	10.8 (96)	0.4 (4)	0 (0)	6 (88)	0.2 (3)	0.6 (9)	8 (100)	0 (0)	0 (0)
5 h	8.6 (0–13)	3.8 (0–5)	13.8 (9–17)	8 (93)	0.6 (7)	0 (0)	2.8 (74)	0.2 (5)	0.8 (21)	13.8 (100)	0 (0)	0 (0)
6 h	7.4 (3–10)	5.8 (0–12)	9 (1–18)	7.4 (100)	0 (0)	0 (0)	4 (69)	0 (0)	1.8 (31)	9 (100)	0 (0)	0 (0)
12 h	3.2 (0–4)	4.2 (0–9)	15.2 (0–28)	0.8 (24)	1.2 (38)	1.2 (38)	1.8 (43)	1.8 (43)	0.6 (14)	13.8 (91)	1.4 (9)	0 (0)
18 h	5.8 (0–8)	11.6 (0–23)	33.4 (0–63)	3 (52)	2.8 (48)	0 (0)	8.6 (74)	2.2 (19)	0.8 (7)	31.4 (94)	1.8 (5)	0.2 (1)
24 h	7 (0–11)	5.2 (0–7)	14.2 (0–22)	0.6 (9)	4 (57)	2.4 (34)	1.6 (31)	0.8 (15)	2.8 (54)	10.6 (75)	3 (21)	0.6 (4)
48 h	0.4 (0–2)	3 (0–15)	5.4 (0–5)	0.4 (100)	0 (0)	0 (0)	0 (0)	0 (0)	3 (100)	1.8 (33)	3.2 (60)	0.4 (7)
72 h	0 (0)	1.6 (0–3)	6.2 (0–9)	0 (0)	0 (0)	0 (0)	0 (0)	0.2 (12)	1.4 (88)	2 (32)	4.2 (68)	0 (0)

^a^Dissected from five infected mosquitoes per time point

## Discussion

The parasites that cause LF must undergo exsheathment to develop further within the mosquito vector. In this study, the exsheathment of *B*. *malayi* microfilariae took place in the midgut of *Ae*. *togoi*, *An*. *lesteri* and *An*. *paraliae* before penetration of the midgut wall. We found that exsheathment of mf in *Ae*. *togoi* started earlier than in the other species and only one sheathed mf was found in the haemocoel of *Ae*. *togoi* at five minutes post-blood meal. We also noted time differences among the studied groups: the exsheathment of mf in the midgut of highly susceptible vectors (*Ae*. *togoi* and *An*. *lesteri*) was around 12 hours, but to complete this process, an additional six hours was required in *An*. *paraliae* (low susceptiblility). Our results agree with previous studies of refractory vectors [19]. Intakhan et al. [19] found that *B*. *malayi* cast off their sheaths only in the midgut of refractory *Ae*. *aegypti* (Thailand strain), beginning within five minutes and continuing over an extended period (48 hours) after the blood meal. Earlier, Nayar & Knight [25] reported that most *B*. *malayi* mf exsheathed in the midgut of *Ae*. *aegypti* (Black eye, Liverpool strain) but some sheathed mf reached the haemocoel and exsheathed there. However, our results contrast with some other studies that describe that mf exsheathed in the haemocoel [20–22, 25, 34, 35]. Christensen & Sutherland [21] used *in vitro* midgut penetration techniques, light and electron microscopy to show that nearly all *B*. *pahangi* microfilariae carried their sheaths into the haemocoel and suggested that their sheaths might break at the anterior end during penetration of the midgut of *Ae*. *aegypti* (Black eye, Liverpool strain). Agudelo-Silva & Spielman [20] revealed that microfilariae penetrated the midgut wall of susceptible *Ae*. *aegypti* (Black eye, Liverpool strain) while still sheathed, and that the sheath remained protruding from the gut into the haemocoel by using scanning electron microscopy. Nayar & Knight [25] showed that the rapid penetration of microfilariae from the midgut to the haemocoel in both susceptible and refractory strains of *An. quadrimaculatus* allowed most of the sheathed microfilariae to carry their sheaths into the haemocoel, then exsheath. However, Chen & Shin [23] showed that some *B*. *pahangi* microfilariae were exsheathed in the midgut of both susceptible (Liverpool) and refractory (Bora-Bora) strains of *Ae*. *aegypti* but that most microfilariae carried their sheaths into the haemocoel within two hours after feeding on infected blood. The activity of chitinase may play a role in the exsheathment of microfilariae. Chitinase of *B. malayi* is a specific enzyme in microfilariae and is found only in the inner body and pharyngeal thread. The chitinase levels found in the excretory/secretory (ES) products collected from exsheathed microfilariae were higher than from non-exsheathed microfilariae [36]. It is possible that chitinase released from *B. malayi* microfilariae could degrade the chitin on the sheath thereby aiding microfilariae escape from the sheath. However, the mechanisms by which microfilariae penetrate the midgut epithelium are unclear, possibly involving mechanical, enzymatic or integrated processes. Esslinger [17] suggested that the sharp projection at the anterior of mf, called the “cephalic hook”, might be used to tear the midgut epithelium during penetration into the haemocoel. This differs from the observation of Agudelo-Silva & Spielman [20] that this structure is blunt and even bulbous. Several enzymes, including glycolytic, proteolytic and lipolytic enzymes, might aid in modifying the midgut epithelium to allow mf to penetrate more easily. Shahabuddin et al. [37] showed that the protease released from the midgut has an adverse effect on *Plasmodium* parasites by increasing at least three-fold the activity of parasitic chitinase which aids in midgut penetration.

Peritrophic matrix (PM) formation, relative to that of mf midgut penetration, has been considered a potential physical barrier to mf penetration in some vector species [17, 38–40]. Michalski et al. [41] reported that the mosquito midgut is the barrier for infectivity of *Brugia* spp. in *Culex pipiens pipiens*, which inflicts internal and lethal damage to ingested microfilariae. In the present study, the migration of microfilariae to thoracic muscles of both high susceptible mosquitoes, *Ae. togoi* and *An. lesteri*, was more rapid than the low *B. malayi* susceptible *An. paraliae*. However, during 48–72 hours post-blood meal, all exsheathed microfilariae successfully migrated out of the midgut which supports the belief that PM does not serve as a physical barrier to nocturnally *B. malayi* in these three mosquito species and corresponds with previous studies [19, 22, 42]. Kato et al. [42] used RNAi to knock-down chitin synthase and demonstrated that PM does not affect the development of *B. pahangi* or the dissemination of dengue virus as well as infectivity of *Plasmodium gallinaceum* in *Ae*. *aegypti* (Black-eyed Liverpool strain). Also, Jariyapan et al. [22] reported that PM degraded from 24 to 72 hours post-blood meal when digestion was completed.

During the time microfilariae travel through the haemolymph-filled haemocoel, cellular and humoral responses can attack and restrict parasite development. These include encapsulation, melanisation, and immune system peptide production [13]. Additionally, there are some reports that the microfilarial sheath may activate an immune response [14, 24, 25, 43]. The thoracic musculature is the developmental site for *W. bancrofti*, *B. malayi* and *B. pahangi*, and here microfilariae develop to L3 in susceptible mosquitoes. In contrast, in refractory mosquitoes, microfilariae are killed in the midgut or haemocoel, or migrate to the developmental site, but fail to develop, which may be due to a physiological incompatibility that is independent of active immune responses [21]. Melanin pigment is a product synthesised in mosquitoes by a complex biochemical pathway [44]. Accumulation or capsule formation of melanin is generally very specific to the surface parasites, and this melanised microfilariae might not be able to continue their further development due to a lack of nutrition [45]. Besides melanisation, larvae with degenerated tissues have been reported as another defence reaction in *Anopheles* spp., and have been explained to result from direct toxicity [12, 28]. In this study, we observed both defence reactions; melanisation and degeneration of microfilariae throughout 12 time points, and we found that the degeneration of microfilariae in the thoracic musculature is the dominant type for *An*. *lesteri* and *An*. *paraliae.* This event occurred first in the haemocoel and thoracic musculature of these species and was followed by melanisation responses. In contrast, a melanisation response occurred first in the haemocoel of *Ae*. *togoi*, as soon as five minutes post-blood meal, and was followed by a degeneration process. Similar results were also observed in the development of *B*. *malayi* larvae in the thoracic musculature of *Ar. subalbatus* [46]. The authors demonstrated that larvae were first melanised in the haemocoel, whereas the degenerated larvae were observed first and then melanin formation of the larvae followed in the thoracic musculature; they suggested that the defence reactions of *Ar. subalbatus* against this filarial worm differ between the haemocoel and thoracic musculature. Furthermore, although intracellular melanisation occurring in tissues, such as thoracic musculature and Malpighian tubules is not common [26], our study demonstrates that the melanisation of microfilariae does occur in the thoracic musculature of mosquitoes that exhibit both high and low susceptibility to filarial worms.

We hypothesise that the degeneration or direct toxicity to the microfilariae may be due to the effect of some chemical components which are generated during the start of the process of melanisation. Melanisation is a complex reaction of enzymatic and non-enzymatic reactions generating melanin pigment. By-products from this process are cytotoxic molecules, including reactive oxygen intermediates (ROI) and reactive nitrogen intermediates (RNI), such as superoxide anion O_2_^-^, hydrogen peroxide (H_2_O_2_), nitric oxide (NO) and its derivatives, and hydrochloric acid (HOCl^-^), through coordinated activities of nicotinamide adenine dinucleotide phosphate (NADPH) oxidase, superoxide dismutase (SOD), and inducible nitric oxide synthase (NOS) [27]. During melanogenesis, NO that is generated and released and can react with a ROI such as O_2_^-^and H_2_O_2_ to form peroxynitrite (ONOO) and hydroxyl radicals (OH) [47]. The role of NO in killing parasites without melanin deposition has been reported in *Drosophila* infected with a parasitoid wasp [47]. NO could damage both host epithelium and pathogens [48]. Although both *in vitro* and *in vivo* studies of inducible NO demonstrated its direct toxicity towards virtually every tested pathogen, from bacteria (*Escherichia coli* and *Micrococcus luteus* [49]), *Plasmodium* spp. [50, 51] to metazoan parasites, including the trematodes *Schistosoma* spp. [52, 53], no detailed studies on filarial nematodes have been carried out. Further studies are required not only to investigate the role of NO in the host-parasite relationship but also gain fundamental information on mosquito NO. In addition to cytotoxic molecules, other essential factors in the mosquito midgut, such as proteolytic enzymes or pH, may directly damage the cells inside the body of the microfilariae because vacuolated or degenerated worms were observed. Furthermore, Michalski et al. [41] suggested that the mechanism of *Cx*. *p*. *pipiens*-induced midgut damage to *Brugia* spp. microfilariae is not yet clear, but the differential vital staining and protease sensitivity of intact (*Ae*. *aegypti*-derived) and damaged (*Cx. p*. *pipiens*-derived) worms indicate that the *Cx*. *p*. *pipiens* midgut environment breaches the microfilaria cuticle, leading to the death of cells inside the worms.

## Conclusions

The exsheathment, migration and innate immune responses of *Ae*. *togoi*, *An*. *lesteri* and *An*. *paraliae* against infection with NSP *B*. *malayi* were systematically investigated for the first time. The exsheathment of microfilariae occurred in the midgut of all mosquito species. All ingested microfilariae were exsheathed entirely in the midgut before 24 hours post-blood meal. We found that the midgut did not function as a barrier to microfilariae migration from this site to the thoracic musculature for all mosquito species. Two defence reactions; melanisation and degeneration of microfilariae were found in the midgut, haemocoel and thoracic muscle fibres of all mosquito species, regardless of their overall susceptibility. In the mosquito with the lowest susceptibility to *B. malayi*, we observed the highest number of abnormal microfilariae; the mechanism for this observation merits additional study.

## Abbreviations

AL: *Anopheles* *lesteri*
AP: *Anopheles* *paraliae*
AT: *Aedes togoi*
LF: lymphatic filariasis
Mf: microfilariae
PI: post-infection
